# Silent Vertebral Fractures in Elderly Patients: A High Incidence Regardless of Gender and Widespread Vitamin D Deficiency—A Pilot Study in Patients Who Have Suffered a Fracture Elsewhere in the Body

**DOI:** 10.3390/jcm14062009

**Published:** 2025-03-16

**Authors:** Guido Schröder, Steffi S. I. Falk

**Affiliations:** 1 Clinic for Orthopaedics and Trauma Surgery, Sana Hospital Bad Doberan, Academic Teaching Hospital of the University of Rostock, Am Waldrand 1, 18209 Hohenfelde, Germany; 2 Clinic of Trauma, Hand and Reconstructive Surgery, University of Rostock, Schillingallee 35, 18057 Rostock, Germany; steffi.falk@med.uni-rostock.de

**Keywords:** silent vertebral fractures, osteoporosis, vitamin D deficiency, low-energy trauma

## Abstract

**Background:** The present study aims to investigate the influence of systematic radiological screening for silent vertebral fractures (VFs) on osteoporosis diagnosis, treatment decisions, and long-term clinical outcomes compared to standard care without routine screening in elderly patients hospitalised primarily for fractures requiring surgical treatment at sites other than the spine. **Material/Methods:** In a level 1 trauma centre, patients with fractures requiring surgical treatment after low-energy trauma were prospectively examined over a period of 12 months. Using radiographs of the thoracic and lumbar spine in two planes, previously unknown VFs were identified and categorised according to the classification for osteoporotic fractures (OFs) of the thoracolumbar spine. **Results:** A total of 106 patients with a mean age of 79.4 years participated in this study, and 112 previously unknown vertebral compression fractures were diagnosed in 57% (60/106) of the patients. In this group, lumbar vertebra 2 was the most frequently affected, and the majority of these VFs were classified as OF 2, which corresponds to an isolated endplate fracture with minimal involvement of the posterior wall. Furthermore, 26% (28/106) of the patients in the evaluation showed VFs at multiple levels. This study revealed no statistically significant difference in the prevalence of silent VFs between male and female patients (*p* = 0.055). Additionally, the analysis revealed that nearly 75% of patients exhibited vitamin D insufficiency. **Conclusions:** The high prevalence of silent VFs in elderly patients emphasises the necessity for systematic radiological investigations, irrespective of gender.

## 1. Background

Osteoporosis (OP) is defined as a bone with reduced mass and functional quality, which leads to an increased risk of fracture with a painless course of the disease until the first fracture. The prevalence of OP in the European Union, the United Kingdom, and Switzerland is 5.6% (22.1% in women; 6.6% in men) [1]. Women are considered to be the affected population group when OP is mentioned. The existing literature on the subject predominantly focuses on women [2,3]. According to the findings of several studies, women have a lower peak bone mass than men and experience age-related bone loss 10 years earlier than men [4]. This was also very clearly reflected in the 2018 guideline of the German Scientific Osteological Societies (DVO), which recommended screening women 10 years earlier than men [5]. The 2018 guideline on OP acknowledged the under-recognition of vertebral fractures (VFs), emphasising the necessity for radiological evaluation in cases of chronic back pain or acute loss of two centimetres in height [5].

The consequences of VFs for those affected are far-reaching. They are associated with health and economic burdens, increased morbidity and mortality, and impaired quality of life [6]. According to the Fracture Risk Assessment Tool (FRAX) [7] or the German guideline for OP [2], previous OP-associated VFs increase the risk of further VFs and thus lower the threshold for drug therapy. In addition, knowledge of pre-existing VFs influences the choice of OP drug therapy. This knowledge is instrumental not only in determining the choice of medication but also, in particular, in deciding whether osteoanabolic therapy is indicated. For illustration, the current German OP guideline [2] from 2023 calculates the fracture risk for a patient for the next 3 years, determining a higher risk with an existing grade 3 VF according to Genant [8] than with a fresh proximal femur fracture [2]. This also incorporates knowledge that the proximal femur fracture has the highest mortality among fractures [9,10,11]. Recent data demonstrate an expanding treatment gap for OP in Germany and Europe. The results of the SCOPE study indicate that this discrepancy has widened, increasing from an average of 54% in 2010 to 74% in the present day [1]. This increase is associated with rising healthcare costs and an increase in the incidence of OP-related fractures. These fractures carry a significant risk of long-term care needs and a consequent impairment in patients’ quality of life. It is also noteworthy that VFs represent a substantial proportion of OP-associated fractures.

Despite the crucial role of VFs in research and clinical practice, there is a paucity of studies addressing the prevalence of silent VFs [12,13]. In particular, there is a lack of literature on the incidence of silent VFs in patients with other OP-related fractures. One of the most important reasons for systematic screening is the fact that osteoporotic VFs often go undetected [14]. The early detection of previously unknown VFs could help to reduce the risk of subsequent fractures and initiate early medical and physical therapy.

The aim of this pilot clinical study was to identify previously unknown silent VFs and distribution patterns in elderly patients with fractures requiring surgery after low-level trauma and to evaluate their implications for OP diagnostics and treatment decisions. This study thus sought to test the following hypotheses:

- Silent VFs are prevalent in this patient population;
- Women exhibit a higher incidence of silent VFs compared to men;
- The incidence of silent VFs increases with age;
- Vitamin D deficiency is more prevalent among patients with silent VFs.

## 2. Material and Methods

### 2.1. Study Design and Group Allocation

The present study design corresponds to a prospective, monocentric, clinical study of an intervention group. Group allocation was based on two factors: firstly, newly diagnosed VFs and, secondly, gender.

### 2.2. Recruitment and Ethics

Patients were recruited for the present study over a period of 12 months, beginning in November 2022 in a level 1 trauma centre of the University Medical Centre. All study participants provided written consent to participate. The conduct of this study was reviewed and approved by the responsible regional ethics committee for medical research (No. A 2022-0102).

### 2.3. Inclusion and Exclusion Criteria

Patients requiring surgical treatment for fractures at other body sites following low-energy trauma were included. Examples of such trauma include a medial femoral neck fracture following a fall over the edge of a carpet. Due to logistical constraints, this study was conducted exclusively on hospitalised fracture patients. This approach ensured the allocation of sufficient time for the organisation of the planned examinations and interviews of the patient. Furthermore, it focused this study on the most vulnerable patient demographic. Surgical fracture treatment is indicated for more severe injuries or when there is a functional advantage to surgical treatment, provided that the patient is sufficiently fit for anaesthesia. The decision to include only patients with fractures requiring surgical treatment is also based on the method of creating a homogeneous patient population. This procedure allows a clearer delimitation of the study population and reduces the variability in the course of treatment. At the same time, it increases the internal validity of this study and enables more precise statements to be made about the investigated correlations. Patients had to be aged at least 45 years. Exclusion criteria included severe bone diseases such as tumours, bone metastases, Paget’s disease, renal insufficiency requiring dialysis, the presence of growth retardation, and relevant anatomical deformities.

### 2.4. Medical History, Laboratory, and Imaging Diagnostics

The study participants underwent a detailed medical history, clinical examination, and laboratory screening. As part of the medical history, existing risk factors and previously known VFs were recorded in accordance with the guidelines for surgical diagnostics. X-rays of the thoracic and lumbar vertebral column were then taken in two planes. 

**Figure 1:**
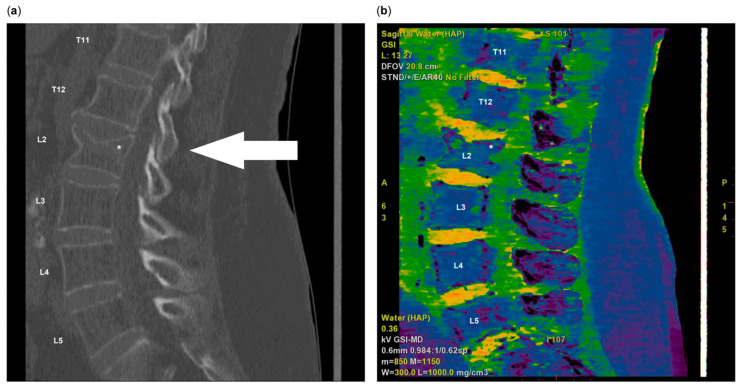
Computed tomography (**a**) and the corresponding dual-energy image (**b**) of a previously unknown L2 vertebral fracture in a 79-year-old female patient. The presence of yellow colouring is indicative of increased fluid content, which is characteristic of the ligamentous disc or oedema observed in acute fractures. Conversely, the homogeneous blue colouring of the vertebral body is an indication that the fracture is a old one. The arrow denotes the vertebral body that has been affected, whilst the asterisk indicates the location of the endplate fracture.

The X-rays of the spine were taken in two planes according to the standard projections in lateral and anterior–posterior view, taking into account the guideline of the German Medical Association for quality assurance in X-ray diagnostics [15]. For each study participant, the image was numbered and the lateral image was labelled with a study identification number. The images were arranged in random order without accompanying clinical information. In the first step, the radiographs were reviewed by two certified and experienced radiologists to identify the fractures. In cases where a VF was identified, high-resolution spiral computed tomography (CT) (Figure 1a) with dual-energy imaging (Figure 1b) was performed to ensure precise detection. The use of two different X-ray energies in this manner facilitates a more accurate differentiation of tissue types. Furthermore, dual-energy CT has been shown to enhance the detection of bone marrow oedema, which can be indicative of a recent fracture, and to demonstrate superior sensitivity in the detection of such fractures when compared with conventional CT [16]. In a secondary, independent step, a second review of the X-ray images was carried out in the Department of Trauma Surgery. Previously identified VFs were not analysed; instead, VFs were categorised according to Genant [8], and OFs (osteoporotic fractures of the thoracolumbar spine) [17] were categorised by an experienced trauma surgeon. The laboratory tests included the recommended parameters of the DVO guideline [2] valid at the initiation of this study. These included a complete blood count, electrolytes (including phosphate and magnesium), creatinine, C-reactive protein, alkaline phosphatase, gamma-glutamyltranspeptidase, thyroid-stimulating hormone, 25-hydroxyvitamin D, and serum protein electrophoresis. The data collected were also used to determine the fracture risk for the next 10 years using FRAX [7]. In order to circumvent the potential for bias resulting from the heterogeneity of fracture entities that had led to hospitalisation, the FRAX risk value was calculated without taking into account the current fracture and any silent VF seen. The decision as to whether the patient had osteoporosis was based on the treatment threshold of 3% for the FRAX risk value for proximal femur fractures and 20% for major osteoporotic fractures [18,19].

### 2.5. Statistics

This pilot study analyses the incidence of VFs and their associated risk factors in an elderly patient cohort. Based on the available data from the literature, in particular the study by Gehlbach et al. [14], a prevalence of silent VFs of about 14% in elderly women was assumed. This study was designed to last for a period of 12 months as a pilot study to record unknown VFs; therefore, no concrete power analysis could be carried out in the run-up to this study in the case of previously unknown incidence. In order to estimate the necessary group size for the subgroup analysis, an a priori calculation was performed using an effect size of Cohen’s d = 0.8 and an alpha error of 0.05. This calculation yielded a minimum sample size of 84 patients in total, equating to 42 patients per group in a two-group comparison.

All data collected were analysed using the statistical software package SPSS (version 28.0, Armonk, NY, USA: IBM Corp., Armonk, NY, USA). Quantitative characteristics were described as the mean (M), standard deviation (SD), and *n* of available observations; they were presented using the interval mean ± standard deviation (M ± SD). The Wilcoxon–Mann–Whitney test and the chi-square test were used for comparisons between the groups, with the relevant choice being made depending on the result of the Kolmogorov–Smirnov test for a normal distribution. All *p*-values are the result of two-sided statistical tests, and in principle *p* < 0.05 is considered significant.

## 3. Results

This pilot study represents the data collected in a level 1 trauma centre over a period of one year. Data from a total of 106 study participants were analysed. The mean age of the patients in this study was 79 years (range: 50–96 years) (Table 1). The study population included 87 female patients (82%) and 19 male patients (18%). The mean age of the female patients was 79.23 years (±10.54 years), and that of the male patients was 79.53 ± 8.11 years, resulting in a gender ratio of 4:1 (*p* > 0.05), with no statistically significant difference in age between the two groups (*p* > 0.05). This study revealed that four women and one man had a history of previous spine fractures, while twenty-seven women and one man were diagnosed with osteoporosis. Of these, five women were receiving OP therapy with antiresorptives.

Table 2 shows the fractures that led to the patient’s admission for male and female patients.

**Figure 2:**
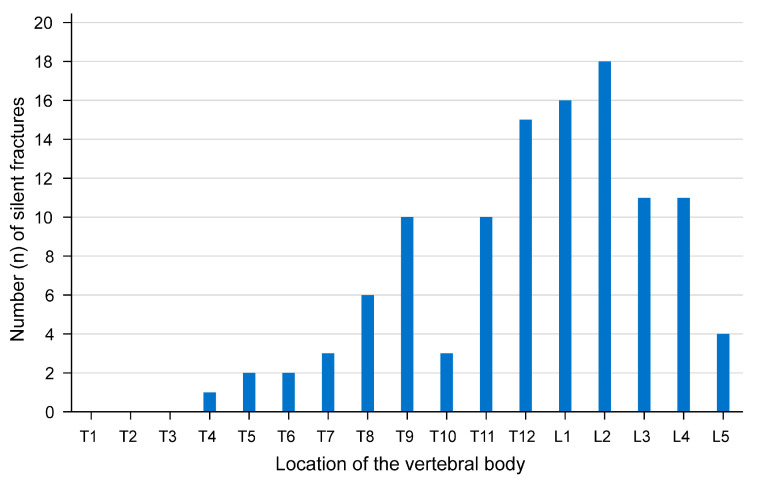
An illustration of the distribution of newly diagnosed vertebral compression fractures according to the affected vertebral body localisation.

**Figure 3:**
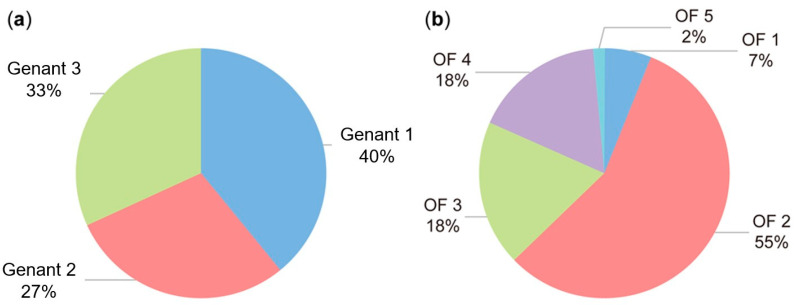
An illustration of the distribution of fracture entities according to the most common classifications for osteoporotic fractures defined by Genant [8] (**a**) and the DGOU [17] (**b**).

The X-ray images of the thoracic and lumbar spine revealed 112 previously unknown VFs in 57% (60/106) of the patients, with 28 patients showing VFs at multiple levels. One patient showed a total of seven VFs of which she and her family were previously unaware. The classification according to Genant [8] shows an almost even distribution of the fractures across the three classes, with a slight tendency towards simple endplate fractures. The lumbar vertebra 2 was the most frequently affected by a fracture (Figure 2). According to the classification system for osteoporotic fractures of the thoracolumbar spine developed by the German Society for Orthopaedics and Trauma Surgery (DGOU) [17], the majority of fractures were classified as isolated endplate fractures with minimal involvement of the posterior wall (Figure 3), and thus categorised as class 2 VFs. Of the 60 patients examined with VFs, 14 were diagnosed with OP.

The distribution of VFs between sexes was examined, and no significant difference was observed (*p* = 0.055). Additionally, no significant difference was found in the study of existing multiple VFs between men and women (*p* = 0.162). Furthermore, the investigation revealed no statistically significant difference in age between the subclasses of patients with (80.4 ± 8.9 years) and without fractures (78.6 ± 9.8) (*p* = 0.531) (Table 3).

The analysis of the vitamin D supply indicates an undersupply irrespective of gender and the presence of previously unidentified VFs. A comprehensive analysis of the values is provided in Table 4. A detailed breakdown of the serum vitamin D values according to the recommendation for deficiency at values below 75 nmol/L reveals that within the group without silent VFs, 76% do not reach this limit. In the group with proven fractures, this falls to 74%. A further breakdown by gender reveals that 73% of women and 81% of men fail to reach the recommended limit of 75 nmol/L. This observed discrepancy is not statistically significant (*p* > 0.05). In addition, there was no association between low vitamin D levels and an increased incidence of VFs.

## 4. Discussion

### 4.1. Frequency of Silent VFs

This study addresses the issue of silent VFs in patients hospitalised due to low-energy trauma with a fracture in another part of the body. Osteoporotic VFs occur every 22 s worldwide in men and women over 50 [20]. This study is particularly relevant as silent VFs often remain undetected and can have a significant impact on the treatment of OP and fracture risk. It is estimated that only one-third of VFs receive clinical attention [21]. The present study found that 57% of the women analysed had silent VFs, which is an alarming finding. Furthermore, the incidence of VFs increases with age in both sexes and is greater in older women than in men. The proportion of VFs not detected during local assessment of a lateral chest X-ray ranges from 46% in Latin America to 45% in North America and 29% in Europe/South Africa/Australia [22]. Less than 10% of VFs result in hospitalisation, even if they cause pain and a significant loss of quality of life [23,24]. A study of adults over the age of 50 in Vietnam reported an incidence of 6.6 new asymptomatic VFs per 1000 person-years over a two-year period [13]. In addition, researchers from Canada found that 16% of patients over 60 years of age had moderate-to-severe VFs on chest X-rays, 40% of which were not documented in the official radiological findings [25]. In Europe, the age-standardised incidence of morphometric fractures is 10.7 and 5.7 per 1000 person-years in women and men, respectively [26]. These figures increase significantly with age in both sexes. According to a study of postmenopausal women on glucocorticoid therapy, over 37% had asymptomatic VFs, with more than 14% having two or more asymptomatic VFs [27]. Furthermore, Majumdar et al. [25] estimate that 12–25% of people aged 50–60 years have suffered one or more osteoporotic VFs, with approximately 70% of VFs being asymptomatic and not becoming clinically apparent. These results underscore the necessity for a systematic investigation of silent VFs, particularly in older women who already suffer from OP or are taking medication that leads to the weakening of the bone substance.

The majority of studies on the prevalence of VFs suggest that the incidence is similar or even greater in men than in women up to the age of 50 or 60 years but slightly greater in women than men thereafter [28,29]. El Maghraoui et al. [12] reported a fracture prevalence of 42% in a group of postmenopausal women, with an average age of 61 years. In Europe, the prevalence, defined by radiological criteria, increases with age in both sexes and is almost equivalent in men and women (12% in women [range of 6–21%] and 12% in men [range of 8–20%]) [29]. This disparity could be explained by occupational trauma in men [30].

A 10% loss of bone mass in the vertebrae has been demonstrated as a factor capable of doubling the risk of developing a vertebral compressive fracture [31].

### 4.2. Gender Distribution and Risk Factors

This study established that the incidence of silent VFs does not significantly vary between men and women, thereby contradicting the prevailing assumption that women have a higher susceptibility to OP-associated VFs. In contrast, Felsenberg et al. [26] documented a higher prevalence of VFs in women within the general population in their epidemiological study. The Tromsø study [32] investigated the prevalence of vertebral fractures in the Norwegian population and found an overall prevalence of 11.8% in women and 13.8% in men, which increased with age from about 3% in the group under 60 years to about 20% in the group over 70 years. Wedge vertebral fractures were the most common in both sexes and occurred mainly in the thoracic spine. Contrary to expectations, the prevalence of vertebral fractures was not higher in Norway than in other populations, although the country has one of the highest incidences of hip and forearm fractures in the world. The European Prospective Osteoporosis Study (EPOS) [33] demonstrated that women exhibited a twofold elevated risk of VFs in comparison to men following age adjustment. In the Rotterdam Study [34], the incidence of vertebral fractures in women increased from 7.8/1000 person-years (55–65 years) to 19.6/1000 person-years (>75 years). In contrast, the incidence of vertebral fractures in men was between 5.2 and 9.3/1000 person-years.

With regard to the prevalence of VFs, Nguyen et al. [13] found a higher percentage in men than in women (14.8% vs. 10.6%). The present study suggests that gender is not the primary factor determining the risk of silent VFs, which calls for a re-evaluation of the risk factors for OP and VFs.

### 4.3. Vitamin D Deficiency

A further critical observation is the prevalence of vitamin D deficiency among the study participants, a phenomenon that was anticipated based on prior research [35]. In the present study, 73% of the female participants did not attain the recommended vitamin D level of 75 nmol/L. These findings are consistent with those reported by Thomasius et al. [5], who observed a correlation between vitamin D deficiency and elevated fracture risk in elderly individuals. The potential consequences of this deficiency include the development of OP and the incidence of silent VFs. It is therefore crucial that physicians consider the vitamin D supply when treating elderly patients with fractures.

### 4.4. Methods to Reduce Intra- and Interobserver Variability in the Assessment of VFs

The reliable and reproducible assessment of VFs represents a diagnostic challenge not only in the present study. The semi-quantitative method according to Genant et al. [8] is one of the most commonly used approaches. This method is based on a visual assessment of vertebral body height and shape and has proven to be extremely reliable. Genant et al. [8] show high intraobserver agreement (93–99%) and interobserver agreement (94–99%). A key factor in reducing interobserver variability is the experience and training of the assessors [8]. Interobserver variability can be significantly reduced by appropriate training. In addition, the use of standardised examination protocols is another important aspect. This includes written protocols with clear VF definitions that are detailed enough to allow the assessments to be reproduced by other experts.

### 4.5. Clinical Implications

The high prevalence of silent VFs and vitamin D deficiency emphasise the need for routine radiological examination in older patients with fractures. The guidelines of the German Scientific Osteological Societies (DVO) [2] already recommend radiological examination for chronic back pain and loss of height, and these recommendations should be increasingly integrated into clinical practice in order to identify silent fractures at an early stage. Early detection of silent VFs can reduce the risk of further fractures. With regard to the early detection of previously unknown VFs, the question of the sensitivity of the radiological examination methods used also arises. In his study, Sheridan et al. [36] compared the sensitivity of reformed CT images with conventional radiographs in trauma patients with thoracic and lumbar spine injuries and showed that the sensitivity of CT imaging was 97% for thoracic and 95% for lumbar spine fractures, compared with 62% and 86%, respectively, for conventional radiographs. With this knowledge, CT could be used to confirm the early diagnosis of a VF and possibly reduce additional radiation exposure if the patient has corresponding symptoms in the thoracic spine. Hedderich et al. [16] specifically investigated the detection rates of VFs by CT and MRI as well as the possibility of identifying fresh VFs in CT. The results show that CT alone had a sensitivity of 0.93 and specificity of 0.88 for fracture detection, while STIR-MRI alone achieved a sensitivity of 0.96 and specificity of 0.70. For the identification of fresh VFs, CT showed a sensitivity of 0.72 and a specificity of 0.98. This study demonstrates that CT is not only reliable in the detection of fractures, but can also distinguish between fresh and older fractures with high specificity.

Furthermore, knowledge of existing silent VFs can be pivotal in determining drug therapy, particularly with regard to osteoanabolic therapy. Li et al. [37] conclude from their systematic literature review that OP medications and supplements are generally cost-effective in men with OP. Screening strategies and fracture follow-up programmes also showed economic benefits in men. The cost-effectiveness and thresholds for intervention were generally similar in the studies conducted in men and women, although the incremental cost-effectiveness ratio was slightly higher in men.

The identification of risk factors, such as vitamin D deficiency, can contribute to the prevention of OP and its consequences. Conversely, the performance of routine radiological examinations is associated with costs, particularly in healthcare systems with limited resources. There is a risk of overdiagnosis and associated unnecessary treatment for patients without significant symptoms or complaints [38]. Furthermore, radiological examinations can be uncomfortable for older patients and may cause additional stress. On the other hand, the data from Rühling et al. [39] suggest that opportunistic use of CT imaging, already performed for other reasons, may be a cost-effective method to screen for OP and potentially prevent VFs. 

### 4.6. Limitations

The findings presented here pertain to a relatively small group of patients. The sample size of 106 patients (87 female, 19 male) actually led to a reduced statistical power. However, the achieved sample size is sufficient for the main question of this study on the overall prevalence of silent VFs and is considered acceptable in the context of a pilot study with consecutive patient recruitment. In contrast, the subgroup analysis regarding the frequency of silent VFs between the genders and their outcome must be critically scrutinised due to the imbalance in sample size. However, they ultimately align with the results reported by El Maghraoui et al. [12], who observed VFs in 40% of women in a postmenopausal group with an average age of 61 years. Consequently, a higher prevalence of VFs can be anticipated in older populations. It should be noted that this study’s participants were exclusively fracture patients, resulting in a pronounced gender disparity, with women constituting the majority of the sample. This selection bias precludes the drawing of definitive conclusions about the broader population; however, it allows for the precise identification of patients with a high probability of manifesting OP due to a fracture. Consequently, it is imperative to ascertain the presence of VFs in this patient group, as it can influence the therapeutic approach, namely whether to pursue an osteoanabolic or osteoclast-inhibiting therapy. A further limitation is the resilience of the medical history, which is used to determine whether the VF is new. This is subject to inaccuracies, as not every patient knows all their illnesses, and relatives are sometimes unable to provide full information. However, this situation also reflects the real situation in emergency departments and outpatient practices, where the decision for treatment is made.

## 5. Conclusions

This clinical pilot study demonstrates that more than 50% of fracture patients had previously unrecognised VFs. The high incidence of these fractures, in conjunction with the prevalence of vitamin D deficiency, underscores the necessity for enhanced diagnostics and therapeutic approaches in older patients with fractures and a risk of OP. The results of this study demonstrate the efficacy of an X-ray examination of the lumbar spine in two planes prior to conducting a dual-energy X-ray absorptiometry (DXA) measurement, thereby enhancing the interpretation of measurement data. Furthermore, this study underscores the significance of X-ray spine diagnostics, particularly in elderly patients, in evaluating risk factors for OP, as silent VFs exert a substantial influence on the therapeutic approach and selection of medication. Future studies should focus on investigating the long-term effects of silent VFs on quality of life and fracture risk, as well as developing effective prevention strategies. In addition, larger cohorts with a balanced gender distribution should be examined in order to be able to statistically prove a possible gender difference.

## Figures and Tables

**Table 1:** The characteristics of the entire study population.

Characteristics	Male (*n* = 19)	Female (*n* = 87)	Total (*n* = 106)
Age (years), M ± SD	79.5 ± 8.1	79.2 ± 10.5	79.3 ± 9.9
Aids, *n* (%)			
None	12 (63.2%)	53 (60.9%)	65 (61.3%)
Walking sticks	4 (21.1%)	7 (8.0%)	11 (10.4%)
Rollator	3 (15.8%)	22 (25.3%)	25 (23.6%)
Wheelchair	0 (0%)	3 (3.4%)	3 (2.8%)
Other	0 (0%)	2 (2.3%)	2 (1.9%)
Living environment, *n* (%)			
Living alone	9 (47.4%)	35 (40.2%)	44 (41.5%)
With partner	4 (21.1%)	24 (27.6%)	28 (26.4%)
With children	6 (31.6%)	17 (19.5%)	23 (21.7%)
Assisted living	0 (0%)	3 (3.4%)	3 (2.8%)
Nursing home	0 (0%)	8 (9.2%)	8 (7.5%)
Degree of care, *n* (%)			
None	13 (68.4%)	63 (72.4%)	76 (71.7%)
1–2	3 (15.8%)	16 (18.4%)	19 (17.9%)
3–5	3 (15.8%)	8 (9.2%)	11 (10.4%)
Nursing service, *n* (%)			
With	6 (31.6%)	27 (31.0%)	33 (31.1%)
Without	13 (68.4%)	56 (64.4%)	69 (65.1%)
No data	0 (0%)	4 (4.6%)	4 (3.8%)
Body mass index, M ± SD	25.52 ± 3.53	24.76 ± 4.58	24.9 ± 4.41
Grip strength (kg) right, M ± SD	25.35 ± 10.70	16.46 ± 6.63	18.36 ± 8.45
Activity compared to peers, *n* (%)			
Worse	4 (21.1%)	14 (16.1%)	18 (17.0%)
Equal	11 (57.9%)	36 (41.4%)	47 (44.3%)
Better	4 (21.1%)	35 (40.2%)	39 (36.8%)
No data	0 (0%)	2 (2.3%)	2 (1.9%)
Sports activity, *n* (%)			
Yes	8 (42.1%)	32 (36.8%)	40 (37.7%)
No	11 (57.9%)	53 (60.9%)	64 (60.4%)
No data	0 (0%)	2 (2.3%)	2 (1.9%)
Comorbidities, *n* (%)			
Diabetes	3 (15.8%)	24 (27.6%)	27 (25.5%)
Rheumatism	0 (0%)	13 (14.9%)	13 (12.3%)
Medication, *n* (%)			
PPI intake	6 (31.6%)	26 (29.9%)	32 (30.2%)
Glucocorticoid intake	2 (10.5%)	7 (8.0%)	9 (8.5%)
Nutritional status (MNA), *n* (%)			
Normal	17 (89.5%)	58 (66.7%)	75 (70.8%)
Risk of malnutrition	2 (10.5%)	20 (23.0%)	22 (20.8%)
Malnutrition	0 (0%)	7 (8.0%)	7 (6.6%)
No data	0 (0%)	2 (2.3%)	2 (1.9%)

The data are expressed as the number (*n*) of available observations and their percentage proportion (%) in addition to information on age, body mass index, and hand grip strength as the mean (M) ± standard deviation (SD).

**Table 2:** The distributions of fracture occurrences within the total sample.

Fracture Type	Male	Female	Total
Proximal humerus	1 (1)	16 (13)	17 (14)
Distal radius	-	18 (10)	18 (10)
Pelvis	3 (1)	2 (2)	5 (3)
Proximal femur	6 (1)	20 (13)	26 (14)
Femoral neck	9 (4)	31 (15)	40 (19)
Total	19 (7)	87 (53)	106 (60)

The number of patients (*n*) with silent vertebral fractures seen in the respective fracture type is indicated in brackets.

**Table 3:** The characteristics of the study population divided into subgroups according to the presence of a silent vertebral fracture.

Characteristics	Without Vertebral Fracture		With Vertebral Fracture	
	Male (*n* = 11)	Female (*n* = 32)	Male (*n* = 8)	Female (*n* = 55)
Age (years), M ± SD	78.3 ± 9.1	78.9 ± 10.4	81.3 ± 6.7	79.5 ± 10.7
Aids, *n* (%)				
None	6 (54.5%)	17 (53.1%)	6 (75.0%)	36 (65.5%)
Walking sticks	4 (36.4%)	4 (12.5%)	0 (0%)	3 (5.5%)
Rollator	1 (9.1%)	8 (25.0%)	2 (25.0%)	14 (25.5%)
Wheelchair	0 (0%)	2 (6.3%)	0 (0%)	1 (1.8%)
Other	0 (0%)	1 (3.1%)	0 (0%)	1 (1.8%)
Living environment, *n* (%)				
Living alone	4 (36.4%)	10 (31.3%)	5 (62.5%)	25 (45.5%)
With partner	3 (27.3%)	9 (28.1%)	2 (25.0%)	18 (32.7%)
With children	4 (36.4%)	9 (28.1%)	1 (12.5%)	5 (9.09%)
Assisted living	0 (0%)	0 (0%)	0 (0%)	3 (5.5%)
Nursing home	0 (0%)	4 (12.5%)	0 (0%)	4 (7.3%)
Degree of care, *n* (%)				
None	7 (63.3%)	18 (56.3%)	13 (68.4%)	32 (58.2%)
1–2	2 (18.2%)	8 (25.0%)	3 (15.8%)	15 (27.3%)
3–5	2 (18.2%)	4 (12.5%)	3 (15.8%)	7 (12.7%)
No data	0 (0%)	2 (6.3%)	0 (0%)	1 (1.8%)
Nursing service, *n* (%)				
With	3 (27.3%)	9 (28.1%)	3 (37.5%)	18 (32.7%)
Without	8 (72.7%)	22 (68.8%)	5 (62.5%)	34 (61.8%)
No data	0 (0%)	1 (3.1%)	0 (0%)	3 (5.5%)
Body mass index, M ± SD	25.34 ± 3.98	24.40 ± 4.70	25.75 ± 3.07	24.97 ± 4.55
Grip strength (kg) right, M ± SD	25.51 ± 9.67	17.30 ± 5.09	25.10 ± 12.8	16.36 ± 7.03
Activity compared to peers, *n* (%)				
Worse	3 (27.3%)	8 (25.0%)	1 (12.5%)	6 (10.9%)
Equal	6 (54.5%)	14 (43.8%)	5 (62.5%)	22 (40.0%)
Better	2 (18.2%)	9 (28.1%)	2 (25.0%)	26 (47.3%)
No data	0 (0%)	1 (3.1%)	0 (0%)	1 (1.8%)
Sports activity, *n* (%)				
Yes	3 (27.3%)	12 (37.5%)	5 (62.5%)	20 (36.4%)
No	8 (72.7%)	19 (59.4%)	3 (37.5%)	34 (61.8%)
No data	0 (0%)	1 (3.1%)	0 (0%)	1 (1.8%)
Comorbidities, *n* (%)				
Diabetes	2 (18.2%)	10 (31.3%)	1 (12.5%)	11 (20.0%)
Rheumatism	0 (0%)	5 (15.6%)	0 (0%)	6 (10.9%)
Medication, *n* (%)				
PPI intake	3 (27.3%)	6 (18.8%)	3 (37.5%)	14 (25.5%)
Glucocorticoid intake	2 (18.2%)	2 (6.25%)	0 (0%)	5 (9.1%)
Nutritional status (MNA), *n* (%)				
Normal	9 (81.8%)	20 (62.5%)	8 (100%)	38 (69.1%)
Risk of malnutrition	2 (18.2%)	8 (25.0%)	0 (0%)	12 (21.8%)
Malnutrition	0 (0%)	3 (9.4%)	0 (0%)	4 (7.3%)
No data	0 (0%)	1 (3.1%)	0 (0%)	1 (1.8%)

The data are expressed as the number (*n*) of available observations and their percentage proportion (%) in addition to information on age, body mass index, and hand grip strength as the mean (M) ± standard deviation (SD).

**Table 4:** An overview of the average vitamin D serum levels (nmol/L) categorised by gender and the presence of a silent vertebral fracture.

	Male	Female	Total
Patients with a vertebral fracture	51.88 ± 25.34	57.70 ± 44.67	54.79 ± 36.31
Patients without a vertebral fracture	55.14 ± 30.85	48.24 ± 34.80	51.69 ± 32.88

The data are expressed as the mean (M) ± standard deviation (SD).

## Data Availability

The data presented in this study are available upon request from the corresponding author.

## Abbreviations

CT	Computed tomography
DGOU	German Society for Orthopaedics and Trauma Surgery
DVO	Guideline of the German Scientific Osteological Societies
DXA	Dual-energy X-ray absorptiometry
EPOS	European Prospective Osteoporosis Study
FRAX	Fracture Risk Assessment Tool
IBM	International Business Machines Corporation
M	Mean
*n*	Number
nmol/L	Nanomoles per litre
No.	The number
OF	Classification for osteoporotic fractures
OP	Osteoporosis
SD	Standard deviation
STIR-MRI	Short Tau Inversion Recovery–Magnetic Resonance Imaging
USA	United States of America
VF	Vertebral fracture
VFs	Vertebral fractures
